# Chronic myelomonocytic leukemia with *NPM1* mutation or acute myeloid leukemia?

**DOI:** 10.1093/oncolo/oyae246

**Published:** 2024-09-30

**Authors:** Sandra Castaño-Díez, José Ramón Álamo, Mònica López-Guerra, Marta Gómez-Hernando, Inés Zugasti, Carlos Jiménez-Vicente, Francesca Guijarro, Irene López-Oreja, Daniel Esteban, Paola Charry, Víctor Torrecillas, Lucia Mont-de Torres, Albert Cortés-Bullich, Álex Bataller, Ares Guardia, Daniel Munárriz, Esther Carcelero, Gisela Riu, Ana Triguero, Natalia Tovar, Dolors Vela, Silvia Beà, Dolors Costa, Dolors Colomer, Maria Rozman, Jordi Esteve, Marina Díaz-Beyá

**Affiliations:** Hematology Department, Hospital Clínic Barcelona, Barcelona, Spain; Institut d’Investigacions Biomèdiques August Pi i Sunyer (IDIBAPS), Barcelona, Spain; Universitat Barcelona, Barcelona, Spain; Hematopathology Section, Pathology Department, Hospital Clínic Barcelona, Barcelona, Spain; Institut d’Investigacions Biomèdiques August Pi i Sunyer (IDIBAPS), Barcelona, Spain; Hematopathology Section, Pathology Department, Hospital Clínic Barcelona, Barcelona, Spain; Centro de Investigación Biomédica en Red de Cáncer (CIBERONC), Madrid, Spain; Hematopathology Section, Pathology Department, Hospital Clínic Barcelona, Barcelona, Spain; Hematology Department, Hospital Clínic Barcelona, Barcelona, Spain; Hematology Department, Hospital Clínic Barcelona, Barcelona, Spain; Institut d’Investigacions Biomèdiques August Pi i Sunyer (IDIBAPS), Barcelona, Spain; Universitat Barcelona, Barcelona, Spain; Hematopathology Section, Pathology Department, Hospital Clínic Barcelona, Barcelona, Spain; Hematopathology Section, Pathology Department, Hospital Clínic Barcelona, Barcelona, Spain; Hematology Department, Hospital Clínic Barcelona, Barcelona, Spain; Hematology Department, Hospital Clínic Barcelona, Barcelona, Spain; Universitat Barcelona, Barcelona, Spain; Universitat Barcelona, Barcelona, Spain; Hematology Department, Hospital Clínic Barcelona, Barcelona, Spain; Hematology Department, Hospital Clínic Barcelona, Barcelona, Spain; Institut d’Investigacions Biomèdiques August Pi i Sunyer (IDIBAPS), Barcelona, Spain; Universitat Barcelona, Barcelona, Spain; Josep Carreras Leukemia Research Institute, Badalona, Spain; Hematology Department, Hospital Clínic Barcelona, Barcelona, Spain; Hematology Department, Hospital Clínic Barcelona, Barcelona, Spain; Pharmacy Service, Hospital Clínic Barcelona, Barcelona, Spain; Pharmacy Service, Hospital Clínic Barcelona, Barcelona, Spain; Hematology Department, Hospital Clínic Barcelona, Barcelona, Spain; Hematology Department, Hospital Clínic Barcelona, Barcelona, Spain; Hematology Department, Hospital General de Granollers, Granollers, Spain; Institut d’Investigacions Biomèdiques August Pi i Sunyer (IDIBAPS), Barcelona, Spain; Hematopathology Section, Pathology Department, Hospital Clínic Barcelona, Barcelona, Spain; Centro de Investigación Biomédica en Red de Cáncer (CIBERONC), Madrid, Spain; Institut d’Investigacions Biomèdiques August Pi i Sunyer (IDIBAPS), Barcelona, Spain; Hematopathology Section, Pathology Department, Hospital Clínic Barcelona, Barcelona, Spain; Centro de Investigación Biomédica en Red de Cáncer (CIBERONC), Madrid, Spain; Institut d’Investigacions Biomèdiques August Pi i Sunyer (IDIBAPS), Barcelona, Spain; Hematopathology Section, Pathology Department, Hospital Clínic Barcelona, Barcelona, Spain; Centro de Investigación Biomédica en Red de Cáncer (CIBERONC), Madrid, Spain; Institut d’Investigacions Biomèdiques August Pi i Sunyer (IDIBAPS), Barcelona, Spain; Hematopathology Section, Pathology Department, Hospital Clínic Barcelona, Barcelona, Spain; Hematology Department, Hospital Clínic Barcelona, Barcelona, Spain; Institut d’Investigacions Biomèdiques August Pi i Sunyer (IDIBAPS), Barcelona, Spain; Universitat Barcelona, Barcelona, Spain; Josep Carreras Leukemia Research Institute, Badalona, Spain; Hematology Department, Hospital Clínic Barcelona, Barcelona, Spain; Institut d’Investigacions Biomèdiques August Pi i Sunyer (IDIBAPS), Barcelona, Spain; Josep Carreras Leukemia Research Institute, Badalona, Spain

**Keywords:** chronic myelomonocytic leukemia, *NPM1*, acute myeloid leukemia, CMML, *NPM1*mut CMML, AML, CMML treatment, *NPM1* CMML

## Abstract

The 2022 WHO revision and the ICC classification have recently modified the diagnostic criteria for chronic myelomonocytic leukemia (CMML) and acute myeloid leukemia. However, there is no consensus on whether CMML with *NPM1* mutation (*NPM1*mut) should be diagnosed as AML. Nowadays, it is a subject of discussion because of its diagnostic and therapeutic implications. Therefore, we describe a case of a patient diagnosed with CMML *NPM1*mut and briefly review the literature to highlight the uncertainty about how to classify a CMML with *NPM1* mutation. We emphasize the importance of a comprehensive molecular study, which is crucial to optimize the individualized treatment of patients, enabling them to access targeted therapies.

## Teaser

This brief communication describes the case of a patient diagnosed with chronic myelomonocytic leukemia with NPM1 mutation and briefly review the literature to highlight the uncertainty about how to classify this condition.

There is currently no clear consensus on whether chronic myelomonocytic leukemia (CMML) with *NPM1* mutations (*NPM1*mut) should be diagnosed and treated as acute myeloid leukemia (AML).^1,2^ Here we report the case of a patient who was diagnosed based on morphological, clinical, and molecular data and treated in a risk-adapted manner. The presented case and the ensuing discussion, based on a review of the literature, highlight the uncertainty about how to classify a CMML with *NPM1* mutation, and the need for comprehensive molecular studies.

**Figure 1.**
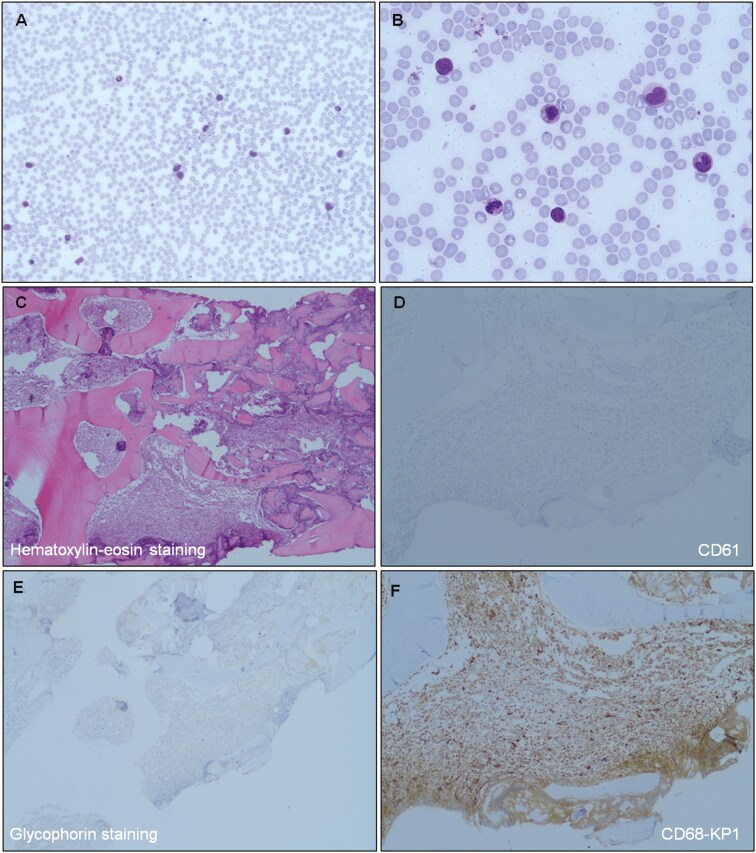
Peripheral blood smear (A, B) and bone marrow biopsy (C-F). The biopsy specimen was highly hypercellular, with a striking increase of granulomonocytic cells (positive for CD68, MPO, and lysozyme), which were shifted toward immaturity. Megakaryocytes were markedly decreased and showed no clustering, with some dysplastic forms. Erythropoiesis was also reduced. There were 10%-15% of CD117-positive immature cells, with no increase in CD34-positive cells. A diffuse loose network of reticulin (MF-1) was observed.

**Figure 2.**
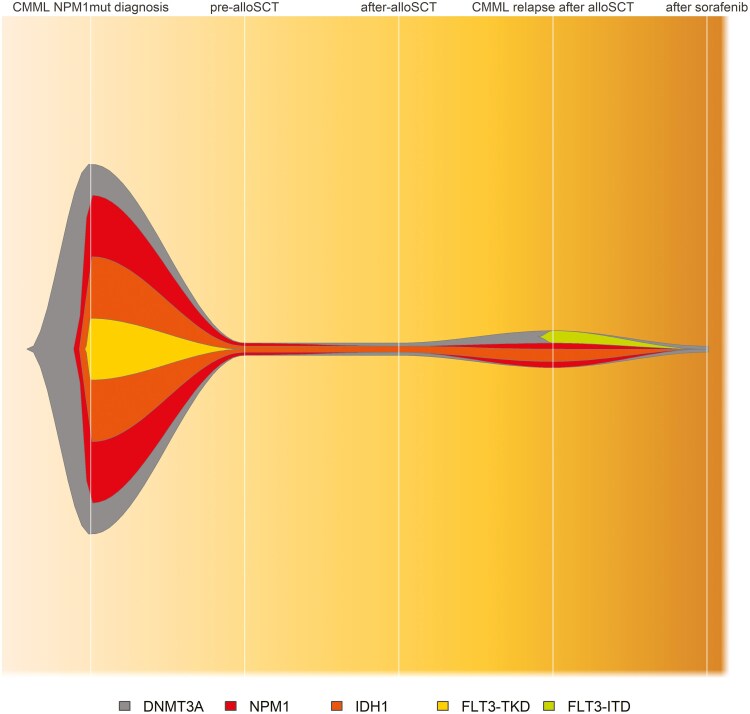
Mutational characterization at different timepoints of patient’s evolution. The targeted NGS panel used was Oncomine Myeloid Research Assay (ThermoFisher Scientific), which comprises the coding regions of 17 genes (*ASXL1*, *BCOR*, *CALR*, *CEBPA*, *ETV6*, *EZH2*, *IKZF1*, *NF1*, *PHF6*, *PRPF8*, *RB1*, *RUNX1*, *SH2B3*, *STAG2*, *TET2*, *TP53*, and *ZRSR2*), the hotspot regions of 23 genes (*ABL1*, *BRAF*, *CBL*, *CSF3R*, *DNMT3A*, *FLT3*, *GATA2*, *HRAS*, *IDH1*, *IDH2*, *JAK2*, *KIT*, *KRAS*, *MPL*, *MYD88*, *NPM1*, *NRAS*, *PTPN11*, *SETBP1*, *SF3B1*, *SRSF2*, *U2AF1*, and *WT1*), and fusion genes of 29 drivers (*ABL1*, *ALK*, *BCL2*, *BRAF*, *CCND1*, *CREBBP*, *EGFR*, *ETV6*, *FGFR1*, *FGFR2*, *FUS*, *HMGA2*, *JAK2*, *KMT2A(MLL)*, *MECOM*, *MET*, *MLLT10*, *MLLT3*, *MYBL1*, *MYH11*, *NTRK3*, *NUP214*, *PDGFRA*, *PDGFRB*, *RARA*, *RBM15*, *RUNX1*, *TCF3*, and *TFE3*).

A 55-year-old woman with no relevant past medical history was diagnosed with CMML type 1, myelodysplastic variant. Three months later, she became transfusion dependent and was referred to our hospital for further management. Complete blood count showed anemia, thrombocytopenia, leukocytosis with monocytosis (15 × 10^9^/L and 2.8 × 10^9^/L), myelemia, and 5% blasts. Dysplasia was seen in the granulocytic and monocytic lineage (Figure 1A, B). Flow cytometry confirmed 5.3% of immature myeloid cells. Karyotype was normal. These findings led us to perform further studies, including a bone marrow aspirate (dry tap) and biopsy (Figure 1C-F). Real-time (RT) PCR did not detect *BCR::ABL1*, *PML::RARA*, and *CBFB::MYH11* rearrangements or *FLT3-*ITD and TKD (D835/I836) mutations, but identified the presence of *NPM1* mutation. Targeted next generation sequencing (NGS) showed 4 pathogenic variants: *DNMT3A* (VAF 38.5%), *FLT3*-TKD (VAF 7.7%), *NPM1* (VAF 38.2%), and *IDH1* (VAF 18.5%) (Figure 2; Table 1).

**Table 1.** Mutational characterization at different timepoints of patient’s evolution.

CMML diagnosis				Pre-alloSCT	Post-alloSCT	CMML relapse				Post-sorafenib
Mutated gene	cDNA	Protein consequence	VAF (%)			Mutated gene	cDNA	Protein consequence	VAF (%)	
*DNMT3A*	c.2645G > A	p.(Arg882His)	38.5	No mutations detected by NGS	No mutations detected by NGS	*DNMT3A*	c.2645G > A	p.(Arg882His)	1.94	No mutations detected by NGS
*FLT3*-TKD	c.2516A > G	p.(Asp839Gly)	7.7			*FLT3-*ITD	c.1802_1803insCGA	p.(Asp593_Leu601dup)	2.7 (ratio 0.028)	
				*NPM1*/*ABL1* ratio 0.023 by qRT-PCR	*NPM1*/*ABL1* ratio 0.023 by qRT-PCR		TTTCAGAGAATATG			*NPM1*/*ABL1* ratio 0 by qRT-PCR
							AATATGATCT			
*NPM1*	c.863_864ins TCTG	p.(Trp288fs)	38.2			*NPM1*	c.863_864ins TCTG	p.(Trp288fs)	2.28	
*IDH1*	c.395G > A	p.(Arg132His)	18.5			*IDH1*	c.395G > A	p.(Arg132His)	2.74	

The final diagnosis was CMML type 2, myeloproliferative variant, with *NPM1*, *FLT3*-TKD, *IDH1*, and *DNMT3A* mutations. CPSS and CPSS-Mol risk stratification was intermediate-2. The patient received induction chemotherapy according to the AML CETLAM-2012 protocol (idarubicin and cytarabine with midostaurin, #NCT04687098), followed by 2 consolidation courses of high-dose cytarabine with midostaurin, and the CMML achieved a complete remission (CR) with a quantitative RT-PCR showing a mut*NPM1*/*ABL1* ratio of 0.023. Subsequently, she received an allogeneic hemopoietic stem cell transplant (allo-HSCT). She later relapsed at 3 months after allo-HSCT, with 10% of blasts and the emergence of a new *FLT3-*ITD mutation; the initial *FLT3*-TKD mutation disappeared. Immunosuppressive treatment was reduced and treatment with sorafenib (an *FLT3*mut inhibitor) was started. Eight months later, the CMML achieved a complete molecular remission (*NPM1* mutation undetectable by qRT-PCR) that lasted for a year. She died 1 year and a half after allo-HSCT. The autopsy identified septic shock as the cause of death and revealed the persistence of CR.

This case highlights the need for an integrated diagnosis of myeloid neoplasms (MN), including not only morphological and clinical data but also several molecular analyses to identify the genetic markers that are essential for an accurate diagnosis and risk-adapted treatment decisions.

The NPM1 gene encodes a chaperone protein crucial for maintaining genomic stability and regulating cellular processes, primarily located in the nucleolus. Mutations in exon 12 result in cytoplasmic localization of the protein, implicating NPM1 in leukemogenesis through diverse mechanisms.^3,4^ NPM1 mutations drive AML in one-third of cases.^4^ They often stem from preexisting clonal hematopoiesis.^4-6^ DNMT3A mutations are considered early molecular events responsible for preceding clonal hematopoiesis. NPM1 mutations are disease-defining genetic lesions serving as gatekeepers for AML,^1,7,8^ whereas FLT3 mutations are considered late events.^9,10^ Although NPM1 mutations are most commonly found in AML, they have also been described in other myeloid neoplasms (MNs), including CMML and myelodysplastic syndrome (MDS). According to the 2022 ICC classification,^2^ the presence of an NPM1 mutation in CMML should be noted but is not sufficient to define de novo AML.^2^ In contrast, the 2022 WHO classification proposes that the detection of NPM1 mutation is sufficient to diagnose AML regardless of the blast count in MDS context, but it does not specify in the CMML context.^1^ A patient with NPM1mut MDS would be diagnosed with AML,^1,2^ but should a patient with NPM1mut CMML with ≥ 10% blasts also be diagnosed with AML? Moreover, according to the 2022 European LeukemiaNet (ELN) recommendations, in patients with a previous history of MDS/myeloproliferative neoplasms (MPN), the diagnosis of CMML should be maintained until ≥20% blasts are observed although AML-type treatment is recommended if ≥10% blasts are detected. A recent study^11^ supported the premise that NPM1 mutations define AML regardless of the blast count, even if <10%, and that patients diagnosed with NPM1mut CMML actually have early-stage NPM1mut AML. Consequently, patients with NPM1mut CMML should be considered for a more intensive, AML-like treatment based on chemotherapy ± allo-HSCT when possible.^5,8,11-18^ Moreover, NPM1 mutations are an ideal target for the assessment of measurable residual disease (MRD) by RT-PCR. This is usually used to monitor MRD in NPM1 mutated AML during treatment and follow-up, to inform clinical decisions such as anticipating hematological relapse.^10,19,20^

Our final diagnosis of CMML was based on clinical characteristics together with bone marrow and peripheral blood findings, although her molecular profile resembled AML more closely than CMML. In this context, NPM1mut AML is frequently associated with DNMT3A and FLT3 co-mutations. In our case NPM1mut CMML is associated with DNMT3A and FLT3 mutations while it is not associated with more typical CMML mutations.^21,22^ Treatment was adapted to both her clinical characteristics (fit for intensive treatment) and her molecular profile. Patients with NPM1mut CMML usually have a poor outcome. Interestingly, it has been described that patients with NPM1mut CMML preserved the chemosensitivity,^12,14,22^ which suggests that fit patients with NPM1mut MN, including CMML, with <20% blasts may benefit more from intensive AML-type chemotherapy than from standard MDS therapies. It is noteworthy that individuals with this condition do not exhibit the favorable prognosis usually seen in de novo NPM1mut AML^22-25^ and new findings suggest a significant clinical progression in the majority of non-acute cases featuring NPM1mut-MNs. This underscores the need for extensive AML-type treatment approaches, even allo-HSCT, whenever possible.^5,8,11-18^ Nonetheless, data on the optimal treatment for these patients are limited and generally based on small retrospective series.^14,15,22,26,27^

Therapeutic innovations are much more developed in AML than in MDS or CMML. In this regard, in a disease like CMML, which currently has such a poor prognosis, it would be important to have access to these new targeted therapy options.^28,29^

The case presented here highlights the importance of including comprehensive molecular assessments both at the initial diagnosis and upon relapse. This underscores the necessity for repeated evaluations, as changes in these mutations have been demonstrated between diagnosis and relapse.^30^ At diagnosis, mutation characterization by NGS allowed us to identify an atypical *FLT3*-TKD mutation that was not detected by RT-PCR, which led us to include midostaurin in the treatment regimen. At relapse, we identified the emergence of an *FLT3*-ITD mutation and therefore introduced sorafenib, an *FLT3* inhibitor that induced a sustained and complete response. *FLT3* is a tyrosine kinase receptor involved in hematopoietic stem cell survival. Approximately 33% of AML harbor activating mutations in the *FLT3* receptor. The majority of *FLT3* mutations consist of internal tandem duplications (ITD), while a smaller portion comprises point mutations within the tyrosine kinase domain (TKD).^31^*FLT3*-ITD mutation becomes a key mechanism for the survival of leukemia stem cells. The standard treatment for patients with *FLT3*-mutated AML is chemotherapy combined with an *FLT3* inhibitor.^31^ In AML post allo-HSCT, the prophylactic use of sorafenib has shown to improve survival.^32,33^ Furthermore, the prevalence and implications of *FLT3* mutation in CMML are currently a matter of discussion. This mutation occurs in fewer than 5% of CMML patients.^34,35^ While it does not automatically signal a progression to AML, its significance lies in its potential impact on therapeutic strategies (*FLT3* inhibitors).^36–40^

In sum, at present there is no consensus between the recent ICC^2^ and WHO^1^ classifications regarding the capacity of *NPM1* mutations alone to define AML, leaving uncertainty whether cases of CMML with *NPM1* mutation should be classified as AML. However, it is plausible that *NPM1*-mutated CMML might indicate the early stages of AML. If fit enough to receive chemotherapy, these patients could benefit from AML-like treatment, which offers more curative potential than standard CMML therapy. An integrated diagnosis based on morphological, clinical, and molecular data is mandatory for the identification of this disease, while treatment should be risk-adapted and rely more on the patient´s characteristics and cytogenetic and molecular profiles than on blast count alone.

## Data Availability

Due to privacy and ethical concerns, the data that support the findings of this study are available on request from the corresponding author.

## References

[1] Khoury JD , Solary E, Abla O, et al. The 5th edition of the world health organization classification of haematolymphoid tumours: myeloid and histiocytic/dendritic neoplasms. Leukemia. 2022;36(7):1703-1719. 10.1038/s41375-022-01613-1 35732831 PMC9252913

[2] Hasserjian RP , Orazi A, Orfao A, Rozman M, Wang SA. The international consensus classification of myelodysplastic syndromes and related entities. Virchows Arch. 2023;482(1):39-51. 10.1007/s00428-022-03417-1 36287260

[3] Grisendi S , Bernardi R, Rossi M, et al. Role of nucleophosmin in embryonic development and tumorigenesis. Nature. 2005;437(7055):147-153. 10.1038/nature03915 16007073

[4] Falini B , Mecucci C, Tiacci E, et al; GIMEMA Acute Leukemia Working Party. Cytoplasmic nucleophosmin in acute myelogenous leukemia with a normal karyotype. N Engl J Med. 2005;352(3):254-266. 10.1056/NEJMoa041974 15659725

[5] Falini B. NPM1-mutated acute myeloid leukemia: new pathogenetic and therapeutic insights and open questions. Am J Hematol. 2023;98(9):1452-1464. 10.1002/ajh.26989 37317978

[6] Grisendi S , Mecucci C, Falini B, Pandolfi PP. Nucleophosmin and cancer. Nat Rev Cancer. 2006;6(7):493-505. 10.1038/nrc1885 16794633

[7] Arber DA , Orazi A, Hasserjian RP, et al. International consensus classification of myeloid neoplasms and acute leukemias: integrating morphologic, clinical, and genomic data. Blood. 2022;140(11):1200-1228. 10.1182/blood.2022015850 35767897 PMC9479031

[8] Prakash S , Arber DA, Bueso-Ramos C, Hasserjian RP, Orazi A. Advances in myelodysplastic/myeloproliferative neoplasms. Virchows Arch. 2023;482(1):69-83. 10.1007/s00428-022-03465-7 36469102

[9] SanMiguel JM , Eudy E, Loberg MA, et al. Cell origin-dependent cooperativity of mutant Dnmt3a and Npm1 in clonal hematopoiesis and myeloid malignancy. Blood Adv. 2022;6(12):3666-3677. 10.1182/bloodadvances.2022006968 35413095 PMC9631557

[10] Falini B , Brunetti L, Sportoletti P, Martelli MP. NPM1-mutated acute myeloid leukemia: from bench to bedside. Blood. 2020;136(15):1707-1721. 10.1182/blood.2019004226 32609823

[11] Falini B , Martelli MP, Brunetti L, Gjertsen BT, Andresen V. The NPM1 mutant defines AML irrespective of blast count. Am J Hematol. 2023;98(7):187. 10.1002/ajh.26946 37119006

[12] Patel SS , Ho C, Ptashkin RN, et al. Clinicopathologic and genetic characterization of nonacute NPM1-mutated myeloid neoplasms. Blood Adv. 2019;3(9):1540-1545. 10.1182/bloodadvances.2019000090 31085507 PMC6517660

[13] Forghieri F , Nasillo V, Paolini A, et al. NPM1-mutated myeloid neoplasms with <20% blasts: a really distinct clinico-pathologic entity? Int J Mol Sci. 2020;21(23):8975. 10.3390/ijms21238975 33255988 PMC7730332

[14] Montalban-Bravo G , Kanagal-Shamanna R, Sasaki K, et al. NPM1 mutations define a specific subgroup of MDS and MDS/MPN patients with favorable outcomes with intensive chemotherapy. Blood Adv. 2019;3(6):922-933. 10.1182/bloodadvances.2018026989 30902805 PMC6436014

[15] Matanes F , AbdelAzeem BMA, Shah G, et al. Chronic myelomonocytic leukemia associated with myeloid sarcomas and NPM1 mutation: a case report and literature review. Ther Adv Hematol. 2019;10(1):2040620719854596. 10.1177/2040620719854596 31217941 PMC6557017

[16] Zhang L , Campion V, Dickson M, Tang C. Acute myeloid leukaemia with NPM1 mutation: no longer having an absolute blast (count). Pathology (Phila). 2023;55(4):578-581. 10.1016/j.pathol.2022.10.009 36707319

[17] Estey E , Hasserjian RP, Döhner H. Distinguishing AML from MDS: a fixed blast percentage may no longer be optimal. Blood. 2022;139(3):323-332. 10.1182/blood.2021011304 34111285 PMC8832464

[18] DiNardo CD , Garcia-Manero G, Kantarjian HM. Time to blur the blast boundaries. Cancer. 2022;128(8):1568-1570. 10.1002/cncr.34119 35133004

[19] Falini B , Dillon R. Criteria for diagnosis and molecular monitoring of NPM1-Mutated AML. Blood Cancer Discov. 2024;5(1):8-20. 10.1158/2643-3230.BCD-23-0144 37917833 PMC10772525

[20] Bataller A , Oñate G, Diaz-Beyá M, et al; Grupo Cooperativo Para el Estudio y Tratamiento de las Leucemias Agudas y Mielodisplasias (CETLAM). Acute myeloid leukemia with NPM1 mutation and favorable European LeukemiaNet category: outcome after preemptive intervention based on measurable residual disease. Br J Haematol. 2020;191(1):52-61. 10.1111/bjh.16857 32510599

[21] Hwang SM , Kim SM, Nam Y, et al. Targeted sequencing aids in identifying clonality in chronic myelomonocytic leukemia. Leuk Res. 2019;84(11):106190. 10.1016/j.leukres.2019.106190 31377458

[22] Vallapureddy R , Lasho TL, Hoversten K, et al. Nucleophosmin 1 (NPM1) mutations in chronic myelomonocytic leukemia and their prognostic relevance. Am J Hematol. 2017;92(10):E614-E618. 10.1002/ajh.24861 28707414 PMC5730408

[23] Courville EL , Wu Y, Kourda J, et al. Clinicopathologic analysis of acute myeloid leukemia arising from chronic myelomonocytic leukemia. Mod Pathol. 2013;26(6):751-761. 10.1038/modpathol.2012.218 23307061

[24] Nie Y , Shao L, Zhang H, et al. Mutational landscape of chronic myelomonocytic leukemia in Chinese patients. Exp Hematol Oncol. 2022;11(1):32. 10.1186/s40164-022-00284-z 35610628 PMC9128105

[25] Ernst T , Chase A, Zoi K, et al. Transcription factor mutations in myelodysplastic/myeloproliferative neoplasms. Haematologica. 2010;95(9):1473-1480. 10.3324/haematol.2010.021808 20421268 PMC2930947

[26] Oki Y , Jelinek J, Beran M, et al. Mutations and promoter methylation status of NPM1 in myeloproliferative disorders. Haematologica. 2006;91(8):1147-1148. 16870548

[27] Caudill JS , Sternberg AJ, Li CY, et al. C-terminal nucleophosmin mutations are uncommon in chronic myeloid disorders. Br J Haematol. 2006;133(6):638-641. 10.1111/j.1365-2141.2006.06081.x 16704439

[28] Chin L , Wong CYG, Gill H. Targeting and monitoring acute myeloid leukaemia with nucleophosmin-1 (NPM1) mutation. Int J Mol Sci. 2023;24(4):3161. 10.3390/ijms24043161 36834572 PMC9958584

[29] Thol FR , Döhner H, Ganser A. How I treat refractory and relapsed acute myeloid leukemia. Blood. 2023;143(1):11-20. 10.1182/blood.2023022481 37944143

[30] Jahn N , Jahn E, Saadati M, et al. Genomic landscape of patients with FLT3-mutated acute myeloid leukemia (AML) treated within the CALGB 10603/RATIFY trial. Leukemia. 2022;36(9):2218-2227. 10.1038/s41375-022-01650-w 35922444 PMC9417991

[31] Bystrom R , Levis MJ. An update on FLT3 in acute myeloid leukemia: pathophysiology and therapeutic landscape. Curr Oncol Rep. 2023;25(4):369-378. 10.1007/s11912-023-01389-2 36808557

[32] Xuan L , Wang Y, Yang K, et al. Sorafenib maintenance after allogeneic haemopoietic stem-cell transplantation in patients with <em>FLT3</em>-ITD acute myeloid leukaemia: long-term follow-up of an open-label, multicentre, randomised, phase 3 trial. Lancet Haematol. 2023;10(8):e600-e611. 10.1016/S2352-3026(23)00117-5 37414062

[33] Burchert A , Bug G, Fritz LV, et al. Sorafenib maintenance after allogeneic hematopoietic stem cell transplantation for acute myeloid leukemia with FLT3-internal tandem duplication mutation (SORMAIN). J Clin Oncol. 2020;38(26):2993-3002. 10.1200/JCO.19.03345 32673171

[34] Itzykson R , Kosmider O, Renneville A, et al. Prognostic score including gene mutations in chronic myelomonocytic leukemia. J Clin Oncol. 2013;31(19):2428-2436. 10.1200/JCO.2012.47.3314 23690417

[35] Lee BH , Tothova Z, Levine RL, et al. FLT3 mutations confer enhanced proliferation and survival properties to multipotent progenitors in a murine model of chronic myelomonocytic leukemia. Cancer Cell. 2007;12(4):367-380. 10.1016/j.ccr.2007.08.031 17936561 PMC2104473

[36] Daver N , Strati P, Jabbour E, et al. FLT3 mutations in myelodysplastic syndrome and chronic myelomonocytic leukemia. Am J Hematol. 2013;88(1):56-59. 10.1002/ajh.23345 23115106 PMC4085099

[37] Hammond D , Montalban-Bravo G. Management and outcomes of blast transformed chronic myelomonocytic leukemia. Curr Hematol Malig Rep. 2021;16(5):405-417. 10.1007/s11899-021-00643-3 34499330

[38] Zhang H , Wang X, Li S, et al. FLT3 amplification as double minute chromosomes in a patient with chronic myelomonocytic leukemia. Dis Markers. 2021;2021(5):9932837. 10.1155/2021/9932837 34194582 PMC8203365

[39] Ramos Perez J , Montalban-Bravo G. Emerging drugs for the treatment of chronic myelomonocytic leukemia. Expert Opin Emerg Drugs. 2020;25(4):515-529. 10.1080/14728214.2020.1854224 33280448

[40] Gu J , Wang Z, Xiao M, et al. Chronic myelomonocytic leukemia with double-mutations in DNMT3A and FLT3-ITD treated with decitabine and sorafenib. Cancer Biol Ther. 2017;18(11):843-849. 10.1080/15384047.2017.1281491 28102729 PMC5710697

